# A Theory for Sparse Event-Based Closed Loop Control

**DOI:** 10.3389/fnins.2019.00827

**Published:** 2019-08-21

**Authors:** Pierre Daye, Sio-Hoi Ieng, Ryad Benosman

**Affiliations:** ^1^StreetLab - Institut de la Vision, Paris, France; ^2^INSERM UMRI S 968, Institut de la Vision, Paris, France; ^3^Sorbonne Universités, UPMC Univ Paris 06, UMR S 968, Institut de la Vision, Paris, France; ^4^CNRS, UMR 7210, Institut de la Vision, Paris, France; ^5^University of Pittsburgh Medical Center, Biomedical Science Tower 3, Pittsburgh, PA, United States; ^6^Robotics Institute, Carnegie Mellon University, Pittsburgh, PA, United States

**Keywords:** dynamic systems, feedback control, control theory, event-based signal processing, level crossing sampling

## Abstract

Most dynamic systems are controlled by discrete time controllers. One of the main challenges faced during the design of a digital control law is the selection of the appropriate sampling time. A small sampling time will increase the accuracy of the controlled output at the expense of heavy computations. In contrast, a large sampling time will decrease the computational power needed to update the control law at the expense of a smaller stability region. In addition, once the setpoint is reached, the controlled input is still updated, making the overall controlled system not energetically efficient. To be more efficient, one can update the control law based on a significant fixed change of the controlled signal (send-on-delta or event-based controller). Like for time-based discretization, the amplitude of the significant change must be chosen carefully to avoid oscillations around the setpoint (e.g., if the setpoint is in between two samples) or an unnecessary increase of the samples number needed to reach the setpoint with a given accuracy. This paper proposes a novel non-linear event-based discretization method based on inter-events duration. We demonstrate that our new method reaches an arbitrary accuracy independently of the setpoint amplitude without increasing the network data transmission bandwidth. The method decreases the overall number of samples needed to estimate the states of a dynamical system and the update rate of an actuator, making it more energetically efficient.

## 1. Introduction

With ever faster and ever cheaper digital computers, the control of dynamic systems has shifted from analog to digital controllers. Critically, the majority of discrete-time control laws assume that the sampling rate of the discretization process is constant. There is currently a discrete-time equivalent for the vast majority of continuous-time control theory principles, from the continuous proportional-integral mechanical “governors” of Maxwell (1867) to the more recent optimal control theories based on Pontryagin's maximum principle (Pontryagin et al., 1962).

The selection of the appropriate sampling rate depends both on the open-loop system dynamics and on the desired dynamics of the controlled system. A system with fast dynamics needs a high sampling rate to ensure the stability of the controlled system at the expense of higher computational power. Moreover, the controlled input of a dynamical system is traditionally updated at each time step independently of the error amplitude. When a controlled system is in a stable configuration at the setpoint, there is obviously no need to sample the data, update the controller and the actuator. Indeed, doing so is not energetically efficient.

In the early 60's, this lack of efficiency combined with lower computational power spurred the development of adaptive discrete-time sampling methods (Dorf et al., 1962; Tomovic and Bekey, 1966). In these methods, an event is sent once the sampled signal increases or decreases by a certain delta (send-on-delta/event-triggered discretization schemes). When the signal doesn't change, there are intrinsically no more updates. More than three decades later, there was a resurgent gain of interest for these discretization methods with aperiodic sampling time leading to new control mechanisms (e.g., Arzén, 1999; Bernhardsson and Aström, 1999; Heemels et al., 2012) for send-on-delta, (Miskowicz, 2005, 2007) for area/integral thresholds, and their analyses, e.g., the effect of noise on the send-on-delta mechanism (Astrom and Bernhardsson, 2002; Cervin and Astrom, 2007). In Hetel et al. (2017), a survey on stability studies of aperiodic sampling systems is provided.

Event-based control mechanisms have also drawbacks. For example, to the best of our knowledge, all current event-based control schemes transmit the signal value within an event. This limits the system dynamic range (ratio between the largest and the smallest value that a signal within a control system can assume) because the data transfer must have the same representation as the system data. Therefore, if one wants to control a system with a high accuracy on a wide range of value, such a control system would need a high bandwidth network to transmit the values of the signal.

In this paper, we propose a new framework and theory for event-based control that are circumventing the problem mentioned above while preserving the benefit of the event-based approach. We formalize a generalized discretization method that stems from the principle of neuromorphic event-based cameras (Posch et al., 2008, 2011) for analog signals. In contrast to traditional frame-based cameras where a clock synchronizes the acquisition of each pixel and the pixels' value is transmitted directly in an image, in event-based cameras each pixel is independent. When a pixel detects a light intensity change of a certain magnitude, it signals the change emitting an event. This event carries information about the time of the change, the position of the pixel and if the light intensity increased or decreased. The principal contribution of this work is to generalize the level-crossing sampling representation in the context of control. We focus on exploiting the benefit provided by a more efficient information coding to reduce computation resources through the use of the duration between two events rather than the value of a signal to update the control law. Therefore, an event can be represented with fewer bits than the input signal and this increases the dynamic range of the control system.

First, we describe a general class of event-based discretization functions and the associated reconstruction process in section 2.1. Then, in section 2.2 we show how uncertainties on the event timing as well as on the initial value of the signal affect the accuracy of the reconstructed signal. In section 2.3, we use a logarithm as discretization function and we show how we tackle the issue of the Zeno phenomenon (Heymann et al., 2005; Lampersky and Ames, 2013). Section 3 presents control results based on the logarithmic discretization. Finally, section 4 concludes the paper.

## 2. Materials and Methods

### 2.1. Non-linear Event-Based Discretization

**Figure 1:**
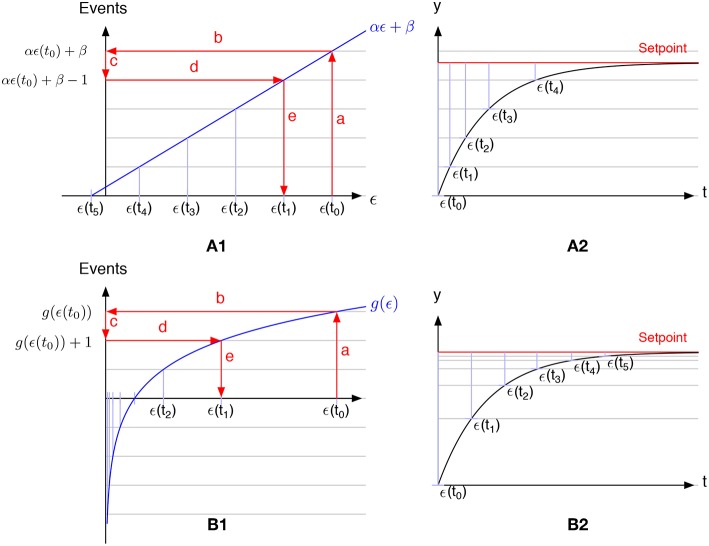
Comparison of linear **(A1)** vs. non-linear **(B1)** event-based discretization. **(A2,B2)** Represent the time course of the controlled system (black lines) and the setpoint (red lines) for the linear and the non-linear discretization. Gray lines represents the levels at which an event will be triggered. Starting from an initial condition ϵ(*t*_0_) (step a), one can evaluate *g*(ϵ(*t*_0_)) (step b). Then remove 1 from the value (step c) to evaluate when the next event will be generated *g*(ϵ(*t*_1_)) = *g*(ϵ(*t*_0_)) − 1. Finally, compute the inverse of *g* for this value and extract the error ϵ(*t*_1_) at the next event (steps d and e). These steps can be repeated to extract ϵ(*t*_2_) from ϵ(*t*_1_), ϵ(*t*_3_) from ϵ(*t*_2_) and so on. As clearly shown in the figure, the limit of this recursive process when the error approaches zero is zero in the non-linear discretization but not in the linear one (because of the bias β in the function).

Figure 1 presents the general principle of a linear (Figure 1A) and a non-linear (Figure 1B) event discretization applied to an error signal ϵ(*t*). Using linear event-based discretization, if the setpoint lies between two intervals then no event will be generated (no event will be generated after *t*_4_ as the system is stable between two samples). To overcome this problem, the simplest method is to compute the step size such that the setpoint is an integer multiple of the step size. It is worth noting that when the setpoint lies between two samples, it corresponds to a bias β in the linear function presented in Figure 1A. In the non-linear case presented in Figure 1B, the step size needed to generate events decreases as the controlled signal approaches the setpoint. Therefore, one can stop the event generation when the absolute error is smaller than a predefined threshold.

#### 2.1.1. Event Generator Function

**Figure 2:**
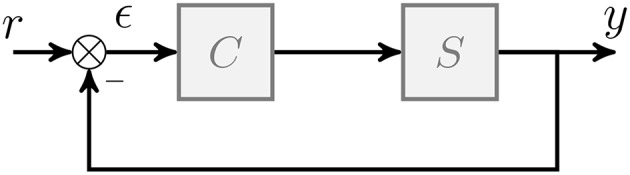
Generic closed loop control system. *r* represents the setpoint, *y* the system output, ϵ the error between the setpoint and the system output, *C* the controller, and *S* the controlled system.

Given a generic finite dimension, non-linear continuous-time system *S* to control, we assume that a control law *C* has been designed to ensure that the output *y* of the system converge toward a setpoint *r*, stabilizing (at least locally) the controlled system. Both signals *r* and *y* can be multidimensional. Using this representation, the error signal ϵ is equal to the difference between the system output *y* and the setpoint *r*. Figure 2 is a schematic summarizing this control configuration.Then, B, a subset of ℝ^+^ computed from the control law *C*, can be defined as the basin of attraction around the equilibrium ϵ = 0 such that:

(1) $$\begin{array}{r} \forall \epsilon \in B\subset {\mathbb{R}}^{*},\underset{t\to +\infty }{\lim}\epsilon \left(t\right)=0, \end{array}$$

(2) $$\begin{array}{r} \underset{t\to +\infty }{\lim}\frac{d\epsilon \left(t\right)}{dt}=0. \end{array}$$

Let *h* be the “event generator” continuous function that computes the time of the next event *t*_*i*+1_ from the value of ϵ at *t*_*i*_.

(3) $$\begin{array}{r} \begin{array}{ccc} h:B & \to & {\mathbb{R}}^{+}\\ \epsilon \left(t_{i}\right) & \mapsto & h\left(\epsilon \left(t_{i}\right)\right)=t_{i+1} \end{array} \end{array}$$

The key principle of our event-based discretization method is that the duration between two events generated by *h* must tend to zero when the error tends to zero. Mathematically, this can be written has a limit:

(4) $$\begin{array}{r} \underset{t\to +\infty }{\lim}\left[\left(h\circ \epsilon \right)\left(t\right)-t\right]=0. \end{array}$$

Equation (4) can be equivalently rewritten into:

(5) $$\underset{t\to +\infty }{\lim}(h\circ \epsilon )(t)=\underset{\epsilon \to 0}{\lim}h(\epsilon )=\underset{t\to +\infty }{\lim}t$$

(6) $$\Rightarrow \underset{\epsilon \to 0}{\lim}h(\epsilon )=\infty .$$

Equation (6) is the first requirement that must be met by *h* if (4) is true.

#### 2.1.2. Events Generation

As the class of functions that satisfy (4) is very broad, this section presents a method to build these functions. One can compute a time series {*t*_*i*_} using a monotonically increasing function *g* applied to ϵ(*t*) such that an event will be triggered when the difference of *g* between two events is equal to one:

(7) $$\begin{array}{r} \forall i\in {\mathbb{N}}^{+}\\ g:{\mathbb{R}}^{+}\to \mathbb{R}\\ \;\left\{t_{i}|\;g\left(\epsilon \left(t_{i-1}\right)\right)-g\left(\epsilon \left(t_{i}\right)\right)=1\right\}. \end{array}$$

For the rest of the paper, we postulate that *g* is monotonically increasing. However, it must be noted that if *g* is monotonically decreasing, the same reasoning can be applied and the time series becomes:

(8) $$\begin{array}{r} \left\{t_{i}|\;g\left(\epsilon \left(t_{i}\right)\right)-g\left(\epsilon \left(t_{i-1}\right)\right)=1\right\}. \end{array}$$

As *g*^−1^ exists (it is monotonically increasing), one can predict the value of the error at which the next event will be triggered:

(9) $$\begin{array}{r} \epsilon \left(t_{i}\right)=g^{-1}\left(\left(g\circ \epsilon \right)\left(t_{i-1}\right)-1\right). \end{array}$$

This discretization process is presented in Figure 1.

In addition, ϵ^−1^ exists by definition of Equation (9). Therefore, one can write the function *h* defined in (4) as:

(10) $$\begin{array}{r} t_{i}=\epsilon^{-1}\left(g^{-1}\left(\left(g\circ \epsilon \right)\left(t_{i-1}\right)-1\right)\right) \end{array}$$

(11) $$\begin{array}{r} =h\circ \epsilon \left(t_{i-1}\right). \end{array}$$

From this relationship, a supplementary condition on *g* can be extracted from (6):

(12) $$\begin{array}{r} \underset{t\to +\infty }{\lim}\left(g\circ \epsilon \right)\left(t\right)=\underset{t\to +\infty }{\lim}\left(g\circ \epsilon \right)\left(t\right)-1. \end{array}$$

As *g* is a function of reals, only ±∞ is a solution of (12).

#### 2.1.3. Signal Reconstruction

Using the proposed discretization, one can reconstruct the time course of the original error signal using the events:

(13) $$\begin{array}{rcl} \epsilon \left(t_{N}\right) & = & \epsilon \left(t_{0}\right)+\overset{N}{\underset{i=1}{\sum }}[g^{-1}\left(\left(g\circ \epsilon \right)\left(t_{i-1}\right)-1\right)\\ \; & - & \epsilon \left(t_{i-1}\right)]H\left(t-t_{i}\right) \end{array}$$

in which *H*(*x*) represents the Heaviside step function:

(14) $$\begin{array}{r} H\left(x\right)=\frac{d}{dx}max\left(x,0\right). \end{array}$$

As the series (13) converges toward 0, then:

(15) $$\begin{array}{r} \underset{N\to +\infty }{\lim}\overset{N}{\underset{i=1}{\sum }}\left[g^{-1}\left(\left(g\circ \epsilon \right)\left(t_{i-1}\right)-1\right)-\epsilon \left(t_{i-1}\right)\right]=-\epsilon \left(t_{0}\right). \end{array}$$

### 2.2. Uncertainty Analysis

Up to now, our formulation of the events generation assumes that there is no measurement uncertainties on both the initial value of the error (ϵ(*t*_0_)) and on the time interval estimation (*t*_*i*_ − *t*_*i* − 1_). In this paragraph, the effect of uncertainties on these values is analyzed.

#### 2.2.1. Uncertainty on the Initial Value

First, we will analyze how an uncertainty on the initial estimate of the error, ϵ(*t*_0_), influences the estimate of ϵ(*t*_*N*_) and thus affects the convergence of the series. We will postulate that we have an infinitely accurate measurement of the time interval between two events.

Given an estimate of the initial error

(16) $$\begin{array}{r} \hat{\epsilon }\left(t_{0}\right)=\epsilon \left(t_{0}\right)+\delta_{\epsilon_{0}} \end{array}$$

with ϵ(*t*_0_) representing the true initial value, δ_ϵ_0__ representing an uncertainty on the true initial value, one can rewrite (13):

(17) $$\begin{array}{rcl} \hat{\epsilon }\left(t_{N}\right) & = & \epsilon \left(t_{0}\right)+\delta_{\epsilon_{0}}\\ \; & + & \overset{N}{\underset{i=1}{\sum }}[g^{-1}\left(g\left(\epsilon \left(t_{i-1}\right)+\delta_{\epsilon_{i-1}}\right)-1\right)\\ \; & - & \epsilon \left(t_{i-1}\right)-\delta_{\epsilon_{i-1}}]H\left(t-t_{i}\right). \end{array}$$

The first terms of recursion (17) are:

(18) $$\begin{array}{r} \hat{\epsilon }\left(t_{0}\right)=\epsilon \left(t_{0}\right)+\delta_{\epsilon_{0}} \end{array}$$

(19) $$\begin{array}{l} \hat{\epsilon }\left(t_{1}\right)=\epsilon \left(t_{1}\right)+\delta_{\epsilon_{1}}\\ =g^{-1}\left(g\left(\epsilon \left(t_{0}\right)+\delta_{\epsilon_{0}}\right)-1\right) \end{array}$$

Because *g* is monotonically increasing, it follows that $\hat{\epsilon }\left(t_{1}\right)$ < $\hat{\epsilon }\left(t_{0}\right)$. This leads to $g^{-1}\left(g\left(\epsilon \left(t_{i-1}\right)+\delta_{\epsilon_{i-1}}\right)-1\right)<\epsilon \left(t_{i-1}\right)+\delta_{\epsilon_{i-1}}$ for arbitrary *i* − 1. Then for *i*, $g^{-1}\left(g\left(\epsilon \left(t_{i}\right)+\delta_{\epsilon_{i}}\right)-1\right)<\epsilon \left(t_{i}\right)+\delta_{\epsilon_{i}}$ is also verified. Therefore, by induction:

(20) $$\begin{array}{r} \underset{N\to \infty }{\lim}g^{-1}\left(g\left(\epsilon \left(t_{i-1}\right)+\delta_{\epsilon_{i-1}}\right)-1\right)=0, \end{array}$$

and

(21) $$\begin{array}{r} \underset{N\to \infty }{\lim}\hat{\epsilon }\left(t_{N}\right)=0. \end{array}$$

This result shows that, independently of the initial error on the measurement, the estimate of the error converges toward zero.

#### 2.2.2. Uncertainties on Time Interval Measurement

In this section, we will analyze the effect of an uncertainty on the measurement of the time interval between two events. If one assumes that the uncertainty σ_*i*_ on the time value *t*_*i*_ is drawn from a uniform random distribution $U_{-\varsigma }^{\varsigma }$ between −ς and ς, one can write Equation (13) as:

(22) $$\begin{array}{rc} \epsilon \left(t\right)+\delta_{\epsilon }\left(t\right) & =\epsilon \left(t_{0}\right)+\overset{N}{\underset{i=1}{\sum }}[g^{-1}\left(\left(g\circ \epsilon \right)\left(t_{i-1}\right)-1\right)\\ \; & \;-\epsilon \left(t_{i-1}\right)]H\left(t-t_{i}+\sigma_{i}\right). \end{array}$$

Using (13) and (22), one can extract δ_ϵ_(*t*), using the rectangular function Π:

(23) $$\begin{array}{r} \Pi \left(X\right)=H\left(X\right)-H\left(0\right) \end{array}$$

(24) $$\begin{array}{r} \delta_{\epsilon }\left(t\right)=\overset{N}{\underset{i=1}{\sum }}\left[g^{-1}\left(\left(g\circ \epsilon \right)\left(t_{i-1}\right)-1\right)-\epsilon \left(t_{i-1}\right)\right]\Pi \left(\sigma_{i}\right). \end{array}$$

From (24), one can evaluate the bounds of the error if one supposes that all the uncertainties are equal to either ς or −ς:

(25) $$\begin{array}{r} \delta_{\epsilon }^{*}\left(t\right)=\pm \varsigma \overset{N}{\underset{i=1}{\sum }}\left[g^{-1}\left(\left(g\circ \epsilon \right)\left(t_{i-1}\right)-1\right)-\epsilon \left(t_{i-1}\right)\right]. \end{array}$$

If ϵ(*t*_0_) = ϵ_0_, the limit of (25) representing the upper (lower) bound of δ_ϵ_(*t*) can be computed using (15):

(26) $$\begin{array}{r} \underset{t\to +\infty }{\lim}\delta_{\epsilon }^{*}\left(t\right)=\mp \varsigma \epsilon_{0}. \end{array}$$

Similarly, the other bound is equal to ςϵ_0_. Equation (26) shows that the boundaries of the uncertainty on the error signal is only a function of the clock accuracy. Therefore, the proposed non-linear discretization can be used to reach an arbitrary precision of the controlled state, provided that the user has access to an infinitely accurate clock.

### 2.3. Logarithmic Event-Based Discretization

As the goal of the paper is to demonstrate the usefulness of the discretization method to control a system, we postulate in the following sections of the paper that the user has designed a control law such that the controlled system is globally asymptotically stable.

In the rest of the paper, we use a logarithmic function as event discretization function *g*(ϵ). We demonstrated in section 2.1 that a candidate discretization function *g* must fulfill three conditions:

1. *g* : ℝ^+,*^ → ℝ
            *x* ↦ *g*(*x*)
2. *g* must be continuous and strictly monotonic on ℝ^+, *^ (and thus invertible)
3. $\underset{x\to 0}{\lim}g\left(x\right)=\left\{ \begin{array}{ccc} -\infty & \text{if} & dg/dx>0\\ +\infty & \text{if} & dg/dx<0 \end{array}\right.$

Selecting a logarithmic function with a base *b* of ϵ(*t*) for *g* such that:

(27) $$\begin{array}{r} \forall b\in {\mathbb{R}}^{+},b\neq 1\\ \;g\left(\epsilon \right)=\frac{ln|\epsilon |}{ln\;b}=log_{b}|\epsilon |, \end{array}$$

it is straightforward to demonstrate that the logarithm satisfies the three conditions on *g* on ℝ^+^. The effect of the base of the logarithm on events generation will be presented in section 3 Using (27), one can compute a time series {*t*_*i*_}:

(28) $$\begin{array}{r} \forall b\in {\mathbb{R}}^{+},b\neq 1,i\in {\mathbb{N}}^{+}\\ \;\left\{t_{i}||\frac{ln|\epsilon \left(t_{i}\right)|}{ln\;b}-\frac{ln|\epsilon \left(t_{i-1}\right)|}{ln\;b}|=1\right\} \end{array}$$

A polarity information, *p*_*i*_, is added to the time series (28) to express if ϵ(*t*) has increased or decreased between two samples of the series:

(29) $$\begin{array}{r} b>1\\ \;\left(t_{i},p_{i}=+1\right)|\left|\frac{\epsilon \left(t_{i}\right)}{\epsilon \left(t_{i-1}\right)}\right|=b \end{array}$$

(30) $$\begin{array}{r} \left(t_{i},p_{i}=-1\right)|\left|\frac{\epsilon \left(t_{i-1}\right)}{\epsilon \left(t_{i}\right)}\right|=b \end{array}$$

For the sake of simplicity, we will use *b* > 1 in the rest of the paper. But the same relationships used to generate an events series for *b* > 1 can be derived if 0 < *b* < 1:

(31) $$\begin{array}{r} 0<b<1\\ \;\left(t_{i},p_{i}=+1\right)|\left|\frac{\epsilon \left(t_{i-1}\right)}{\epsilon \left(t_{i}\right)}\right|=b, \end{array}$$

(32) $$\begin{array}{r} \left(t_{i},p_{i}=-1\right)|\left|\frac{\epsilon \left(t_{i}\right)}{\epsilon \left(t_{i-1}\right)}\right|=b. \end{array}$$

Finally, as ϵ(*t*) can change sign between two events, we added a third piece of information to each event representing a change of sign between the current event and the prior one: *s*_*i*_.

Therefore, an event *e*_*i*_ is defined as a triplet (*t*_*i*_, *p*_*i*_, *s*_*i*_) in which *t*_*i*_ represents the event time, *p*_*i*_ represents its polarity (either 1 or –1, could be represented by a single bit) and a Boolean *s*_*i*_ (0/1) representing the fact that the sign of the signal changed between the *e*_*i*−1_ and *e*_*i*_.

Using these notations, one can construct the time course of the original signal ϵ(*t*) using^1^:

(33) $$\begin{array}{r} \epsilon \left(t\right)=\epsilon \left(t_{0}\right)+{\left(-1\right)}^{s_{N}}\overset{N}{\underset{i=1}{\sum }}\left(b^{-p_{i}}-1\right)\epsilon \left(t_{i-1}\right)H\left(t-t_{i}\right),b>1 \end{array}$$

in which *H*(*x*) represents the Heaviside step function:

(34) $$\begin{array}{r} H\left(x\right)=\frac{d}{dx}max\left(x,0\right). \end{array}$$

#### 2.3.1. Arbitrary Accuracy

The goal of this section is to demonstrate that the proposed logarithmic discretization can be used to reach a stable point with an arbitrary accuracy. Using (29), the value of the error signal at time *t*_*i*_ can be written using a geometric recursion:

(35) $$\begin{array}{r} \epsilon \left(t_{i}\right)=\frac{\epsilon \left(t_{0}\right)}{b^{i}}. \end{array}$$

From (35), it is clear that from any finite value ϵ(*t*_0_):

(36) $$\begin{array}{r} \underset{n\to +\infty }{\lim}\epsilon \left(t_{i}\right)=0. \end{array}$$

Equation (36) shows that one can reach an arbitrary precision using the logarithmic discretization proposed in this section. The arbitrary precision at which the error is considered null (therefore at which the system has reached the setpoint) is a design parameter of the control law.

#### 2.3.2. Refractory Period

Theoretically when ϵ reaches zero, the number of events goes to infinity and the duration between two events reaches zero. In addition, even if the accuracy of the clock is very high, practically the computations needed to evaluate the ratios (29) and (30) can take some time. Therefore, for practical implementations, we define the refractory period as the minimum time between two events that the system can generate. These limitations put some constraints on the overall system as the ratio between the past error and the current one could be crossed multiple times during the refractory period. To counter this issue, we added a last parameter to the event representing the number of times the threshold has been crossed between two events *r*_*n*_:

(37) $$\begin{array}{r} r_{i}=\left\lfloor \frac{ln\left(\epsilon \left(t_{i-1}\right)/\epsilon \left(t_{i}\right)\right)}{ln\;b}\right\rceil \text{if }p_{i}=-1 \end{array}$$

(38) $$\begin{array}{r} =\left\lfloor \frac{ln\left(\epsilon \left(t_{i}\right)/\epsilon \left(t_{i-1}\right)\right)}{ln\;b}\right\rceil \text{if }p_{i}=+1. \end{array}$$

Equation (33) can be written to include *r*_*n*_:

(39) $$\begin{array}{r} \epsilon \left(t\right)=\epsilon \left(t_{0}\right)+{\left(-1\right)}^{s_{N}}\overset{N}{\underset{i=1}{\sum }}\left(b^{-p_{i}r_{i}}-1\right)\epsilon \left(t_{i-1}\right)H\left(t-t_{i}\right),b>1. \end{array}$$

The presented discretization method allows us to benefit from the event-based representation in the control context. In the experiments section, we present its application to the control of a second order dynamical system.

## 3. Experiments

The proposed event-based non-linear discretization and the resulting control laws are tested on two systems different systems. The first one is a classical second order dynamical system while in the second example we stabilize an inverted pendulum on a cart.

### 3.1. Control of a Second Order Dynamical System

This section presents the control of a second order dynamical system with 0.1 and 0.01 s as time constants. The second-order system is controlled by a proportional-integral-derivative controller (see Appendix in Supplementary Material, section 3) with a proportional gain equal to 1, an integral gain equal to 5, and a derivative gain equal to zero. This example is used as an academic demonstrator of the new event-based discretization method. Therefore, this second-order model doesn't describe a particular system and the units of the controlled signals, the setpoint, the output or of any of the system internal signals are arbitrary.

**Figure 3:**
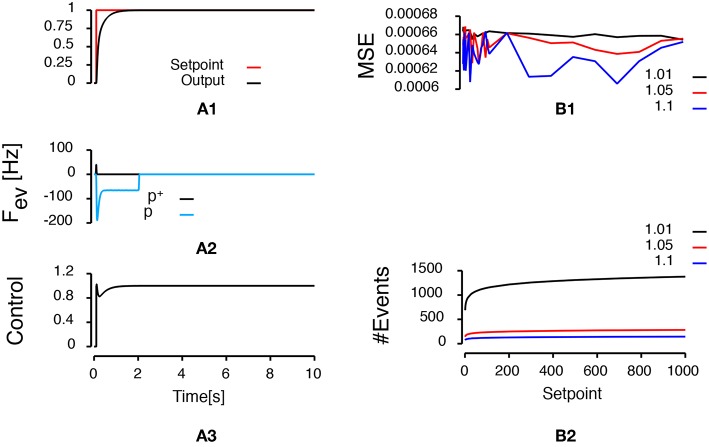
Control of a second-order system. Left column presents the simulation of a second order transfer function controlled by an event-based proportional-integral controller when a step input is applied after 100 milliseconds. **(A1)** Represents the time course of the setpoint (red line) and the time course of the output of the system (black line). **(A2)** Represents the frequency of events as a function of time. Positive frequencies correspond to the frequency of positive polarity events representing an increase of the error signal (*p*^+^: black line). Negative frequencies correspond to the frequency of negative polarity events representing a decrease of the error signal (*p*^−^: light blue line). **(A3)** Represents the time course of the control applied to the system to reach the setpoint. The right column represents the sensitivity of the controlled system to a change of setpoint for different bases (represented by different colors). **(B1)** Represents the evolution of the mean squared error as a function of the setpoint. **(B2)** Represents the evolution of the number of events triggered during a simulation as a function of the setpoint.

Figure 3A presents the results of the simulation when a unitary step (the setpoint of the system goes from zero to one) is applied to the controlled system 100 ms after the beginning of the simulation. The base of the logarithmic discretization function was set to 1.05 and the refractory period was set to 1 millisecond. The upper row represents the time course of the setpoint and the output of the system. The second row represents the frequency of the events generated. Positive (negative) frequencies correspond to positive (negative) polarity events. The events frequency was computed using the convolution of a 2.5 ms normal distribution with the time of the events as it used in neuroscience to compute the discharge frequency of neuronal activities (MacPherson and Aldridge, 1979; Richmond et al., 1987). The last row represents the control sent to the system to reach the setpoint. Importantly, the control is updated only when an event is generated. Figure 3A shows that the output of the system reaches the setpoint. In addition, when the system output reaches the target (around 2 s, final error = 6.48e-4), no event is generated and the control remains constant. 142 events were generated during this simulation.

To test how a choice of base for the logarithmic discretization function affects the control quality, we ran a series of simulations for setpoint amplitude ranging from 1 to 1,000. Upper row in Figure 3B shows the evolution of the mean squared error during the last 250 ms as a function of the setpoint amplitude for three different bases (1.01: black lines, 1.05: red lines, 1.1: blue lines). The average mean squared error is statistically independent of the setpoint amplitude (mean ± standard error of the mean squared error. 1.01: 6.64e-5 ± 5.16e-6, 1.05: 6.56e-5 ± 8.92e-6, 1.1: 6.41e-5 ± 1.52e-5). Lower row in Figure 3B shows that the number of events generated during a simulation for the same conditions decreases when the base of the logarithmic discretization function increases.

This first set of simulations demonstrated that the absolute error remained constant over a wide range of setpoint amplitudes (from 1 to 1,000) without changing either the discretization or the PID parameters. As the absolute error at the end of the simulation remained mostly constant with increasing amplitude, the relative error is decreasing.

### 3.2. Stabilization of an Inverted Pendulum

We have implemented a numerical simulation of an inverted pendulum put on a cart. When a perturbation (in the form of an external force *T*) is applied to the cart, the pendulum is moved out of its equilibrium position. An event-based proportional-integral-derivative (PID) controller stabilizes the pendulum in an inverted position through a control force *F* applied to the cart. The model of this dynamical system is presented in the Appendix in Supplementary Material.

In the first two simulations, we measure the angular orientation of the pendulum. In the last two simulations, we used two sensors to measure the cart position and the angular orientation of the pendulum. In all the simulations, we control the force applied to the cart to stabilize the pendulum in upright position. In each of the following simulations, the pendulum started in the upright inverted position and we injected a fifty milliseconds perturbation one hundred milliseconds after the beginning of the simulation.

#### 3.2.1. Proportional-Integral-Derivative Control Law

In this section, the pendulum angle was controlled by a proportional-integral-derivative (PID) controller with a proportional gain of 100, an integration gain of 2 and a derivative gain of 10. We compared a traditional discrete PID controller with an event-based version of the PID controller. The base of the logarithmic discretization function was set to 1.05 for the event-based control law simulations and the refractory period of the sensor was set to 1 ms.

**Figure 4:**
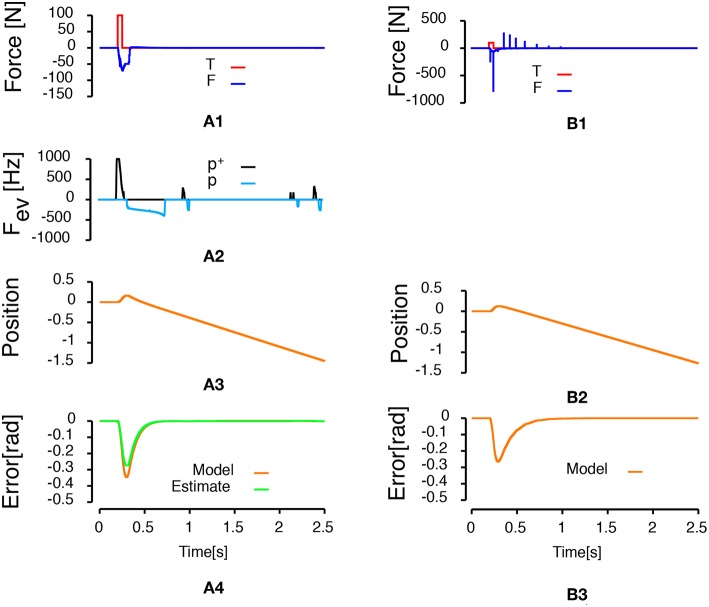
Inverted pendulum on a cart controlled by either an event-based controller (Left column) or a discrete PID with a one millisecond sampling time (Right column). **(A1,B1)** Represent the time course of forces applied to the system. The red curve represents the 100 N perturbation applied to the cart during 50 ms. The blue curve represents the time course of the input force applied to the cart to stabilize the pendulum. **(A2)** Represents the frequency of events generated during the simulation. Positive frequencies correspond to positive polarity events representing an increase of the error signal (*p*^+^: black line). Negative frequencies correspond to negative polarity event representing a decrease of the error signal (*p*^−^: light blue line). **(A3,B2)** Represent the time course of the cart position. **(A4,B3)** Represent the angular error of system (orange line, angle with respect to the vertical) and the estimate of the error built from the received events (green line).

Figure 4 compares the two control laws when the control sampling rate of the discrete controller was set to 1 kHz. For the event-based control law, the control was updated each time an event arrives. Figure 4A shows the results of the simulation with the event-based control law. Figure 4B represents the results of the simulation with the discrete PID. The last row in Figures 4A,B represents the angular error of the pendulum. In both control law conditions, the pendulum is stabilized by the control law. The maximum error is smaller in the case of the discrete PID controller. However, with the discrete PID, there are peaks of desired force above 500 N. In addition, the discrete version of the control law updated the force applied to the cart 2,500 times while the event-based version updated that force only 199 times, so the system is updated roughly 12.5 times less often using the event-based control.

This simulation shows that the frequency of events generation is sensitive to the amplitude of the error (the smaller the error, the higher the number of events) and to the amplitude of the time derivative of the error (the larger the amplitude of the time derivative of the error, the higher the events frequency). In Figure 4, the frequency of positive events increases during the first part of the error increase (when the time derivative of the error is important). Then, as the amplitude of the error time derivative decreases and the amplitude of the error increases, the frequency of events generation decreases as well. Afterwards, there is a small plateau during which no positive nor negative events are generated (corresponding to the peak of the error signal, when there is no modification of the error amplitude large enough to trigger an event). Finally, there is an increase of the frequency of negative events as the error decreases due to feedback control of the PID. It can be seen that, as the rate of change of the error during the decrease of the error is smaller, the overall frequency of the events is smaller. However, even if the time derivative of the error decreases, the events generation frequency increases toward the end of the stabilization period as the error reaches zero. These observations show the sensitivity of the events generation frequency to both the rate of change of the error and the amplitude of the error.

**Figure 5:**
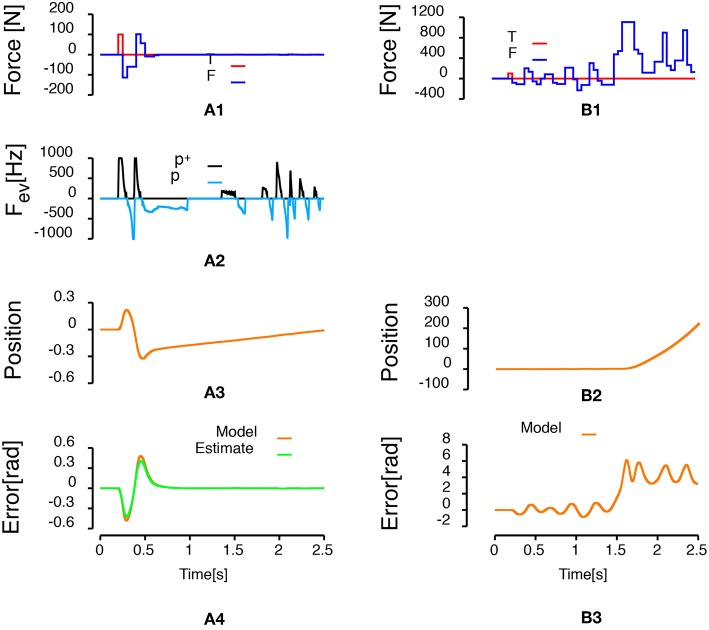
Inverted pendulum controlled with the event-based controller and the discrete controller set to update the control output at 20 Hz. The discrete controller fails to stabilize the pendulum when the control frequency is reduced from 1KHz to 20 Hz. Each subfigures and the colors convention are defined as in Figure 4. **(A1,B1)** Represent the time course of forces applied to the system. The red curve represents the 100 N perturbation applied to the cart during 50 ms. The blue curve represents the time course of the input force applied to the cart to stabilize the pendulum. **(A2)** Represents the frequency of events generated during the simulation. Positive frequencies correspond to positive polarity events representing an increase of the error signal (*p*^+^: black line). Negative frequencies correspond to negative polarity event representing a decrease of the error signal (*p*^−^: light blue line). **(A3,B2)** Represent the time course of the cart position. **(A4,B3)** Represent the angular error of system (orange line, angle with respect to the vertical) and the estimate of the error built from the received events (green line).

Figure 5 compares a discrete PID control law and an event-based control law when the discrete controller and the event-based controller are set to update the control output at 20 Hz. This experiment presents how the event-based controller can be used to update regularly a control output. Therefore, contrarily to all the other experiments, in this case, while the estimate of the error and the integral of the error are updated each time an event is generated, the control is updated every 50 ms. The striking point in Figure 5 is that the event-based controller stabilizes the pendulum (the effect of the external force is negated and the pendulum remains in an upright position) while the system is unstable with the discrete controller. More events are generated in this condition compared to the condition in Figure 4A (432 events here vs. 199 events in Figure 4A).

In this set of simulations, we showed that the system could reject a perturbation applied to the cart and keep the pendulum stable while decreasing the number of times the controlled input is updated by a factor 12 compared to an equivalent time-discrete PID with a 1 ms sample time. We also showed that we can keep the pendulum stable with the same controller parameters while updating the force applied to the cart every 50 ms instead of each time an event is received, reducing drastically the burden put on the actuator.

#### 3.2.2. State-Space Feedback Control Law

**Figure 6:**
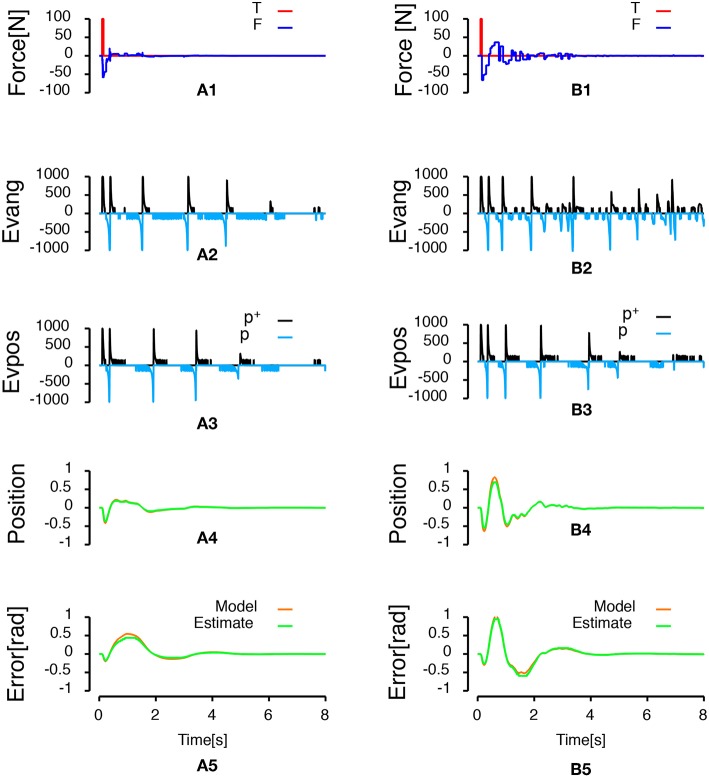
Inverted pendulum controlled by state-space feedback law. Left column represents an event-based state-space feedback law with the force applied to the cart updated at each event. Right column represents the same control law but with the force applied to the cart updated every 50 ms. **(A1,B1)** Show the time course of forces applied to the system. The red curve represents the 100 N perturbation applied to the system during 50 ms. The blue curve represents the time course of the force applied to the cart to stabilize the pendulum. **(A2,A3,B2,B3)** Represent respectively the frequency of pendulum angle and cart position events as a function of time. Positive frequencies correspond to the frequency of positive polarity events representing an increase of the error signal (*p*^+^: black lines). Negative frequencies correspond to the frequency of negative polarity events representing a decrease of the error signal (*p*^−^: light blue lines). **(A4,B4)** Show the time course of the cart position (orange line, deviation from the central position) and the estimate of the position built from the received position events (green line). Finally, **(A5,B5)** Represent the angular error of system (orange line, angle with respect to the vertical) and the estimate of the error built from the received events (green line).

After the controllers reject the perturbation and stabilize the pendulum angle, the third row in Figures 4, 5 shows that the cart keeps moving. This displacement is generated because the stabilized angle is not the upright position. It is not possible to build an observer of the cart position based on pendulum angle measurements as the observability matrix in this condition is not full-rank. Therefore, it is not possible to stabilize both the pendulum angle and the cart position using a single PID with a single sensor on the pendulum angle. In this section, we present a state-space feedback control law that stabilizes both the pendulum angle and the cart position. To that goal, we used two sensors, one on the cart position and one on the pendulum angle. The optimal gain *K* of the state-space feedback law was computed using the linear-quadratic regulator^2^ updated each time an event is emitted by one of the sensors. We used the estimate of the cart position, the derivative of the estimate of the cart position, the estimate of the angular error and the derivative of the estimate of the angular error as states for the feedback. The estimate of the derivatives was built like the derivative component in the PID controller of the previous section. Figure 6A shows the results of the simulation when the force applied to the cart is updated every time one of the sensors emits an event. Figure 6B shows the results of the simulation when the force applied to the cart is updated only every 50 ms. The simulations show that the designed state-space feedback control law rejects the perturbation and that both the cart position and the pendulum angle are stable by the end of the simulations. A total of 1049 events (451 position events, 598 angular events) were generated during the simulation presented in Figure 6A, while a total of 1,679 events (1,113 position events, 566 angular events) were generated during the simulation in Figure 6B.

In this final set of simulations, we stabilized both the inverted pendulum angle and the cart position. The designed system rejected the perturbation and stabilized both the pendulum angle and the cart position. As for the PID simulations, the force applied to cart was updated every 50 ms and we showed that we could keep the system stable.

## 4. Discussion

This work proposes a new non-linear event-based discretization method and the associated proportional-integral-derivative and state-space feedback control laws. The key novelty of the new event generator function lies in the principle that the time between two events must decrease when the discretized signal (e.g., an error signal) tends to zero. Contrary to current event-based discretization/control schemes (e.g., Arzén, 1999; Bernhardsson and Aström, 1999; Miskowicz, 2005, 2007; Tabuada, 2007; Lunze and Lehmann, 2010; Donkers and Heemels, 2012; Heemels et al., 2012), the generated events do not contain the value of the signal. Instead, a transmitted event contains four parts: the time of the change, a polarity (did the signal increased or decreased), a sign changed bit (did the signal's sign changed) and a refractory gain (the number of times the threshold set in the level-sampling mechanism has been crossed during a refractory period) to account for multiple level crossings during the shortest measurable duration between two events.

Using the new event-triggering scheme, we showed that all the uncertainties on the signal approximation emerge from uncertainties on the measurement of the duration between two events. Also, because the inter-event duration is the key information to the signal reconstruction, the components of the system do not require synchronized clocks but all of them must measure a duration with the same accuracy.

In addition, the precision of the representation of the error signal stored in any active part of the control mechanism (e.g., 128 bits to represent the state of the system) can be much higher than the number of bits used to transmit an event (e.g., an 32 bits unsigned integer to represent milliseconds). As a result, the design of a control scheme based on the new method of this paper can increase the controlled signal dynamic range without increasing the network data bandwidth needed to transmit the information between the different components of the controlled system.

Our results demonstrate that the new method combines the advantages of analog continuous time systems

- it can reach an arbitrary precision with a very high dynamic range
- with the advantages of event-based control
- no events are generated when the error is null.

In addition, the amount of data transmitted between active parts of the control system can be smaller than the memory needed to store the signals value. This makes the overall system more efficient energetically.

## Data Availability

All datasets generated for this study are included in the manuscript and the Supplementary Files.

## Author Contributions

PD, S-HI, and RB conceived of the presented idea. PD developed the theory and performed the computations. All authors discussed the results and contributed to the final manuscript.

### Conflict of Interest Statement

The authors declare that the research was conducted in the absence of any commercial or financial relationships that could be construed as a potential conflict of interest.

## Footnotes

^1^If 0 < *b* < 1, $\epsilon \left(t\right)=\epsilon \left(t_{0}\right)+{\left(-1\right)}^{s_{N}}\overset{N}{\underset{i=1}{\sum }}\left(b^{p_{i}}-1\right)\epsilon \left(t_{i-1}\right)H\left(t-t_{i}\right)$

^2^A detailed description of the linear-quadratic regulator is outside the scope of this paper. The interested reader can learn about it in various textbooks (e.g., in Corriou, 2004).
