# Supplementary material for: A Theory for Sparse Event-Based Closed Loop Control
Source: Front Neurosci. 2019 Aug 21;13:827. doi: 10.3389/fnins.2019.00827 (PMC6712166; doi:10.3389/fnins.2019.00827)
Supplement: Supplementary file 1 [file Data_Sheet_1.PDF]

A theory for sparse event-based closed loop control

Pierre Daye [1,*], **Sio-Hoi Ieng** [2], **and Ryad Benosman** [2,3,4]
*Correspondence:
Pierre Daye
pierre.daye@gmail.com

APPENDIX

1 PENDULUM ON A CART DYNAMICAL SYSTEM

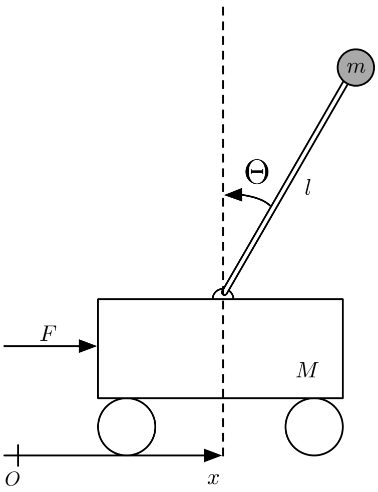

Figure S1. Schematic of the inverted pendulum mounted on a cart.

We consider an inverted pendulum placed on cart. We control the force F applied to the cart and measure only the angle Θ of the pendulum as shown in figure S1. The continuous equations that represents the system are:

$$(M+m)\ddot{x} + m\,l\,(\ddot{\Theta}\,cos\Theta - \dot{\Theta}^2 sin\Theta) = F - \beta\,\dot{x} + \nu \tag{S1}$$

$$m\,l\,cos\Theta\,\ddot{x} + (I + m\,l^2)\ddot{\Theta} + m\,g\,l\,sin\Theta = -\Gamma\,\dot{\Theta}. \tag{S2}$$

In equations (S1) and (S2), F represents the applied force to the system, ν represents a perturbation applied to the cart, x represents the position of the cart, Θ represents the angle of the pendulum on the car, β (Γ) represents the position (angular) viscous friction coefficient, m (M) represents the mass of the pendulum (cart) and I represents the pendulum inertia. All the parameters of the model are presented in Table 1.

Parameter	Symbol	Value
Cart mass	M	1.0
Pendulum mass	m	0.2
Pendulum length	l	0.3
Gravity constant	g	9.81
Pendulum inertia	I	0.006
Cart viscous friction coef.	β	0.1
Pendulum viscous friction coef.	Γ	0.1

Table 1: Parameters of the pendulum on a cart dynamical model.

2 PENDULUM ON A CART LINEARIZED DYNAMICAL SYSTEM

We linearize the system about the setpoint ($\Theta = \pi$). We assume that $\Theta = \pi + \epsilon$ with $\epsilon <<$, eq. (15) and (16) can be written as:

$$(M+m)\ddot{x} - m\,l\,\ddot{\epsilon} = F - \beta\,\dot{x} + \nu \tag{S3}$$

$$(I + m\,l^2)\ddot{\epsilon} - m\,l\,\ddot{x} - m\,g\,l\,\epsilon = -\Gamma\,\dot{\epsilon}. \tag{S4}$$

Equations (S3) and (S4) can be written in a state-space representation:

$$\mathbb{X} = \begin{bmatrix} x \\ \dot{x} \\ \epsilon \\ \dot{\epsilon} \end{bmatrix} \tag{S5}$$

$$\dot{\mathbb{X}} = A\mathbb{X} + B\,(F + \nu) \tag{S6}$$

$$K = Mml^2 + I(M+m) \tag{S7}$$

$$A = \frac{1}{K}\begin{bmatrix} 0 & 1 & 0 & 0 \\ 0 & -(I+ml^2)\beta & m^2l^2g & -ml\Gamma \\ 0 & 0 & 0 & 1 \\ 0 & -\beta ml & mgl(M+m) & -\Gamma(M+m) \end{bmatrix} \tag{S8}$$

$$B = \frac{1}{K}\begin{bmatrix} 0 \\ I+ml^2 \\ 0 \\ ml \end{bmatrix}. \tag{S9}$$

Using the parameters in Table 1, the linearized system in eq. (S6) has 0., -8.194, -0.083 and 3.421 as eigenvalues. The positive eigenvalue confirm that the system is unstable in the upright position. The controllability matrix $[B, BA, BA^2, BA^3]$ is a full-rank matrix, thus the system is controllable through a force applied to the cart.

For the PID simulations, the output matrix is equal to:

$$\Theta = C_p \mathbb{X} \tag{S10}$$

$$C_p = \begin{bmatrix} 0 & 0 & 1 & 0 \end{bmatrix}. \tag{S11}$$

The observability matrix $[C_p^t, A^t C_p^t, A^{2t} C_p^t, A^{3t} C_p^t]$ is not full-rank. Therefore, it is not possible to build an full-state observer using a sensor on the pendulum angle.

For the state-space feedback, the output matrix is equal to:

$$\begin{bmatrix} x \\ \Theta \end{bmatrix} = C_s \mathbb{X} \tag{S12}$$

$$C_s = \begin{bmatrix} 1 & 0 & 0 & 0 \\ 0 & 0 & 1 & 0 \end{bmatrix}. \tag{S13}$$

It is straightforward to see that the observability matrix $[C_s^t, A^t C_s^t]$ is now full-rank. Therefore, it is possible to build an full-state observer using a sensor on the pendulum angle and a sensor on the cart position.

3 PROPORTIONAL-INTEGRAL-DERIVATIVE CONTROLLER

A control output of a proportional-integral-derivative controller using the proposed event-based logarithmic discretization can be written as:

$$u(t_k) = K_p \epsilon(t_k) + K_i I(t_k) + K_d \frac{d\epsilon(t_k)}{dt} \tag{S14}$$

with $I(t_k)$, the integral term.

The derivative term of the controller is computed using a approximated difference:

$$\frac{d\epsilon(t_k)}{dt} \approx \frac{\epsilon(t_k) - \epsilon(t_{k-1})}{t_k - t_{k-1}} \tag{S15}$$

$$\approx \epsilon(t_{k-1}) \frac{(-1)^{s_k}(b^{p_k} - 1)}{t_k - t_{k-1}}. \tag{S16}$$

The integral term is approximated using a trapezoidal approximation:

$$I(t_k) = \int_0^{t_k} \epsilon(\sigma) d\sigma \approx I(t_{k-1}) + \frac{\epsilon(t_k) + \epsilon(t_{k-1})}{2(t_k - t_{k-1})} \tag{S17}$$

$$\approx I(t_{k-1}) + \epsilon(t_{k-1}) \frac{2 + (-1)^{s_k}(b^{p_k} - 1)}{2(t_k - t_{k-1})}. \tag{S18}$$

4 LINEAR-QUADRATIC REGULATOR STATE-SPACE FEEDBACK SENSOR

The state-space feedback control law stabilizes the pendulum angle and the cart position through the computation of the input using a gain K applied to the states of the system:

$$u = -K\mathbb{X}. \tag{S19}$$

We compute the optimal feedback gain K using the LQR method with:

$$Q = \begin{bmatrix} 200 & 0 & 0 & 0 \\ 0 & 0 & 0 & 0 \\ 0 & 0 & 2000 & 0 \\ 0 & 0 & 0 & 0 \end{bmatrix} \tag{S20}$$

$$R = 1 \tag{S21}$$

as parameters. As the LQR method requires a linear representation of the system, we linearized equations (S1)-(S2) around the central upright position. The linearization process is described in the Supporting Information. Briefly, the LQR finds the optimal gain through the minimization of a cost function by solving of a Riccati equation. Describing the LQR is outside the scope of this paper but the interested reader can find more details in numerous textbooks, e.g. in Corriou (2004).

REFERENCES

Corriou, J.-P. (2004). Process control: theory and applications, *Springer*.
